# Identification of Amyotrophic Lateral Sclerosis Based on Diffusion Tensor Imaging and Support Vector Machine

**DOI:** 10.3389/fneur.2020.00275

**Published:** 2020-04-28

**Authors:** Qiu-Feng Chen, Xiao-Hong Zhang, Nao-Xin Huang, Hua-Jun Chen

**Affiliations:** ^1^College of Computer and Information Sciences, Fujian Agriculture and Forestry University, Fuzhou, China; ^2^Department of Radiology, Fujian Medical University Union Hospital, Fuzhou, China

**Keywords:** amyotrophic lateral sclerosis, diffusion tensor imaging, white matter, support vector machine, diagnosis

## Abstract

**Objectives:** White matter (WM) impairments involving both motor and extra-motor areas have been well-documented in amyotrophic lateral sclerosis (ALS). This study tested the potential of diffusion measurements in WM for identifying ALS based on support vector machine (SVM).

**Methods:** Voxel-wise fractional anisotropy (FA) values of diffusion tensor images (DTI) were extracted from 22 ALS patients and 26 healthy controls and served as discrimination features. The revised ALS Functional Rating Scale (ALSFRS-R) was employed to assess ALS severity. Feature ranking and selection were based on Fisher scores. A linear kernel SVM algorithm was applied to build the classification model, from which the classification performance was evaluated. To promote classifier generalization ability, a leave-one-out cross-validation (LOOCV) method was adopted.

**Results:** By using the 2,400~3,400 ranked features as optimal features, the highest classification accuracy of 83.33% (sensitivity = 77.27% and specificity = 88.46%, *P* = 0.0001) was achieved, with an area under receiver operating characteristic curve of 0.862. The predicted function value was positively correlated with patient ALSFRS-R scores (*r* = 0.493, *P* = 0.020). In the optimized SVM model, FA values from several regions mostly contributed to classification, primarily involving the corticospinal tract pathway, postcentral gyrus, and frontal and parietal areas.

**Conclusions:** Our results suggest the feasibility of ALS diagnosis based on SVM analysis and diffusion measurements of WM. Additional investigations using a larger cohort is recommended in order to validate the results of this study.

## Introduction

Amyotrophic lateral sclerosis (ALS) is a cryptogenetic and fatal neurodegenerative disorder that occurs in adults, involving the upper motor neurons as well as lower motor neurons. ALS is a heterogeneous disease and is generally difficult to diagnosis during the early stages. Most ALS patients die of respiratory failure. The median survival time of ALS is 3–5 years (1). Riluzole can only prolong survival time by 2–3 months (2). Therefore, early diagnosis of ALS is particularly important.

Diffusion tensor imaging (DTI) is a non-invasive scanning procedure that provides exquisite details on white matter (WM) tissue microstructure (3) and thus plays a key role in investigating the pathology of neurological disorders. DTI parameters, such as fractional anisotropy (FA), axial diffusivity (AD), radial diffusivity (RD), and mean diffusivity (MD), provide information on the molecular diffusion in various directions. Several DTI studies have revealed that ALS patients have the decreased FA and increased diffusivity parameters in both motor and extra-motor areas (4–6). Of these changes, the alterations in DTI measurements in the corticospinal tract (CST) and corpus callosum are considered as the promising biomarker candidate for the diagnosis and evaluation of ALS (7). For instance, many studies have consistently demonstrated the reduction of CST FA value (6) and its high ability to differentiate ALS patients from healthy controls, at the group-level (8, 9). Also, several discrimination studies at the individual level have suggested that altered diffusion metrics in the CST and corpus callosum can be used as the sensitive marker to identify ALS (10, 11). Furthermore, DTI measurement may have an improved potential role in the correct discrimination of ALS when combined with other neuroimaging biomarkers (such as cortical thickness and functional measurement) (10, 12). In addition, adopting diffusion measuring and high-resolution volumetric/surface imaging simultaneously, researchers have demonstrated the feasibility of multi-model neuroimaging for predicting survival of ALS patients (13, 14).

Recently, the field of machine learning that holds promise to enable the computer-aided diagnosis of neuropsychological disorders has attracted widespread attentions. With the various model neuroimaging data (such as high-resolution T1-weighted image, magnetic resonance spectroscopy, and DTI), many machine learning approaches have been successfully implemented in the predictive modeling of ALS (15, 16). Of them, Random Forests method is a good case in point, which can incorporate multimodal imaging data and achieve quite promising result for individual identification of ALS (10, 12).

Support Vector Machine (SVM) is another algorithm that has been employed in assessing the discriminative brain map of patients diagnosed with ALS (15). In fact, there is increasing studies examining the application of SVM with neuroimaging data for clinical prediction of ALS. For example, a previous resting-state functional magnetic resonance imaging investigation has employed SVM to identify ALS based on the functional connectivity measurements in brain networks and achieved high accuracy for disease state classification (17). Recent studies have also demonstrated that ^18^F-FDG PET (^18^F-2-fluoro-2-deoxy-D-glucose Positron Emission Tomography) with SVM discriminant analysis can yield the promising result in differentiating ALS patients from healthy controls. In this exploratory study, we were to make the attempt to test the potential of voxel-wise diffusion measurements in WM in identifying ALS, based on the SVM learning method.

## Materials and Methods

### Subjects

A total of 22 ALS patients (1 familial, 21 sporadic) as well as 26 healthy controls (HC) were enrolled in this study. We employed the El Escorial criteria (18) in diagnosing ALS, whereas the revised ALS Functional Rating Scale (ALSFRS-R) was utilized to assess their severity of disease. The clinical and demographic information of the study participants are presented in Table 1. No significant differences between the patient and control groups in terms of age, sex, or educational level were observed (the detailed information see Table 1). The exclusion criteria were as follows: (1) existence of other neuropsychiatric disorders, including Parkinson's disease, Alzheimer's disease, epilepsy, or depression; (2) receiving psychotropic drugs; (3) occurrence of respiratory failure or other severe conditions such as angiocardiopathy or cancer; or (4) contraindication of MRI examination. Approval for this evaluation was obtained from the Research Ethics Committee of Fujian Medical University Union Hospital, China. All of the subjects provided their written informed consent.

**Table 1:** Demographic and clinical information of the study participants.

	**Healthy controls (*n* = 26)**	**ALS patients (*n* = 22)**	***P-*value**
Age (years)	53.1 ± 6.4	55.4 ± 6.0	0.21^*^
Sex (male/female)	16/10	15/7	0.82^#^
Education (years)	8.2 ± 3.2	7.5 ± 3.3	0.63^*^
Site of onset (Bulbar/Cervical/Thoracic/Lumbosacral)	−	1/14/1/6	−
Diagnostic category (Definite/Probable/Possible)	−	7/6/9	−
ALSFRS-R score	−	40.1 ± 7.2	−
Disease duration (months)	−	15.7 ± 13.2	−
Disease progression rate	−	0.66 ± 0.49	−

ALS, amyotrophic lateral sclerosis; ALSFRS-R, revised ALS Functional Rating Scale. Calculation of Disease Progression Rate was performed using the equation: (48 - ALSFRS - R)/Disease duration. “-” denotes no data available. P-values marked with “*” and “#” were calculated by the Student's t-test and chi-square test, respectively.

### MRI Data Acquisition

A 3T MRI scanner (Prisma, Siemens Medical Systems, Erlangen, Germany) was utilized in image acquisition. DTI data were gathered with a spin-echo single-shot echo-planar imaging sequence using the following parameters: *b-*value = 1,000 s/mm^2^ and 64 encoding diffusion directions; repetition time = 2,500 ms; echo time = 81 ms; number of averages = 1; slice thickness = 2 mm without gaps; field of view = 260 × 260 mm; matrix = 130 × 130; flip angle = 90°; 72 axial slices; and multiband factor = 4.

### DTI Data Processing

DTI data were processed by an FSL-based pipeline (19). We corrected the raw DTI data for head movement and eddy-current distortion, and we then fitted a diffusion tensor model independently for each voxel. Thereafter, FA images of each subject were obtained. All of the FA images of the study participants were aligned to an FMRIB-58 FA template in Montreal Neurological Institute (MNI) space by a non-linear registration algorithm. Then, all of the images were smoothed with a 6 mm full width at half maximum (FWHM) Gaussian kernel.

### Machine Learning Classification

**Figure 1:**
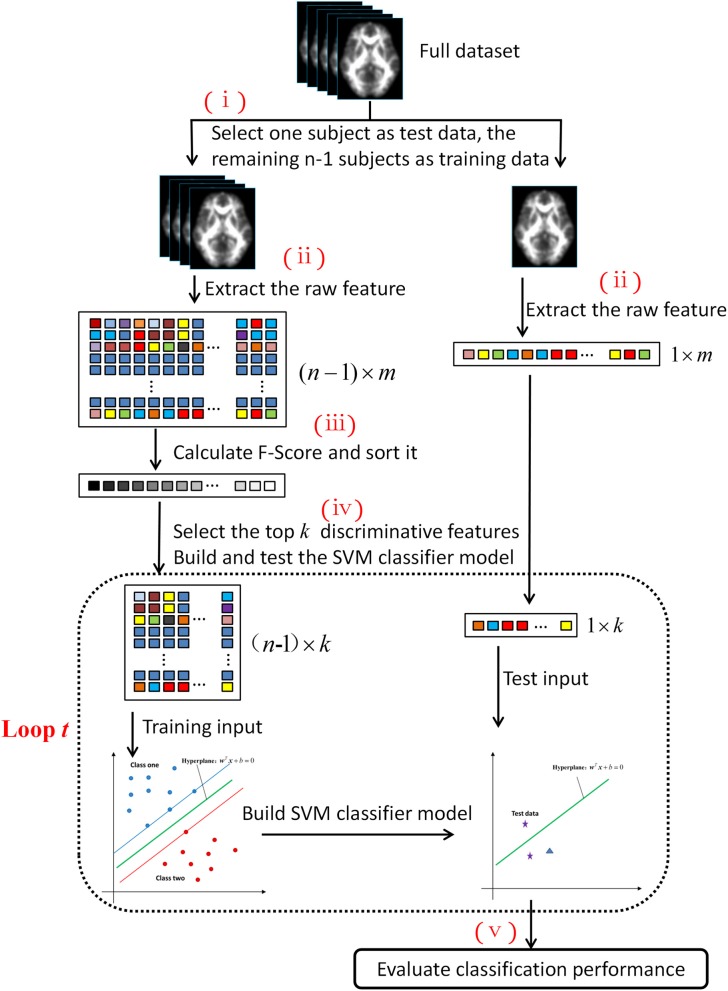
Machine learning flowchart based on the SVM algorithm.

Figure 1 shows the five steps of the machine learning process: (i) splitting the entire dataset into two parts: one subject served as the test set, whereas the remaining comprised the training set; (ii) extracting the features from the DTI images; (iii) calculating the Fisher scores of each feature and ranking the features, (iv) selecting the top *k* discriminative features to build the SVM classifier model and test it; and (v) evaluating the performance of the machine learning classification. The code was implemented in MATLAB (release 2016a, MathWorks, Natick, MA, USA), based on the LibSVM toolbox (20).

To assess the generalization performance of the developed SVM classifier, we adopted the leave-one-out cross-validation (LOOCV) method. If the number of subjects was *n*, then this cross-validation process is conducted *n* times (i.e., the flowchart in Figure 1 is repeated *n* times). In each fold, one subject was selected as the test dataset, and the remaining *n*-1 subjects constituted the training dataset.

In step (ii), the feature vector was extracted from the FA image for each subject to form the raw feature matrixes. The dimensions of the feature matrixes were (*n*-1) × *m* and 1 × *m* for the training and test datasets, respectively.

Due to the presence of the unrelated or redundant features, the learning model will tend to overfit, which in turn will degrade the classification performance (21). The Fisher score algorithm is a supervised feature selection method that can effectively measure data discrimination from two classes and assign the higher score to the feature, which has more discriminative ability (22).

We calculated the score for each feature independently based on the Fisher criterion. Then, we selected the top *k* features with the highest ranking scores. The Fisher score for the *q*th feature is defined as follows:

(1) $$\begin{array}{l} F\left(q\right)=\frac{{\left({\bar{x}}_{q}^{\left(1\right)}-{\bar{x}}_{q}\right)}^{2}+{\left({\bar{x}}_{q}^{\left(2\right)}-{\bar{x}}_{q}\right)}^{2}}{\frac{1}{n1-1}\overset{n1}{\underset{p=1}{\sum }}{\left(x_{p,q}^{\left(1\right)}-{\bar{x}}_{q}^{\left(1\right)}\right)}^{2}+\frac{1}{n2-1}\overset{n2}{\underset{p=1}{\sum }}{\left(x_{p,q}^{\left(2\right)}-{\bar{x}}_{q}^{\left(2\right)}\right)}^{2}}, \end{array}$$

where ${\bar{x}}_{q}$, ${\bar{x}}_{q}^{\left(1\right)}$, and ${\bar{x}}_{q}^{\left(2\right)}$ are the mean values of the *q*th feature of the entire cohort and the HC and patient group, respectively; *n*1 and *n*2 are the number of the HC and patient subjects, respectively; $x_{p,q}^{\left(1\right)}$ denotes the *q*th feature of the *p*th subject in HC group; and $x_{p,q}^{\left(2\right)}$ represents the *q*th feature of the *p*th subject in the patient group. The numerator represents interclass variance, whereas the denominator signifies intraclass variance. Apparently, the greater the Fisher score, the stronger the discriminative power of the feature.

The features were sorted in descending order according to the pre-calculated Fisher scores. Then, some of these features were selected as the input to build and test the SVM classifier model. Figure 1 shows that the dotted box shows a loop within the machine learning process. For each loop, the ranked features were selected with an increase in length of 100 (i.e., for loop *t*, the number of the selected feature (denoted as *k*) was equal to *t* × 100). The total number of cycles was 200; therefore, the length of the selected feature ranged from 100 to 20,000. Feature sorting and selection were built in a nested leave-one-out procedure to promote model generalization, by which we obtained the training input and test input (whose dimensions were (*n*-1) × *k* and 1 × *k*, respectively).

Based on these inputs, the SVM classifier model was trained and tested. The SVM is a classification algorithm that separates two classes by means of the maximal hyper-plane margin. Its main task is to establish a discriminant decision function *f* from the training input, so that for the test input ***x***, this decision function can predict the class label through *y* = *f*(***x***). The decision function is defined in this form:

(2) $$\begin{array}{l} y=f\left(x\right)=w^{\text{T}}\phi \left(x\right)\;+\;b, \end{array}$$

where ***w*** is the weight vector perpendicular to the decision hyper-plane; T represents the matrix transpose manipulation; *b* is the offset of the hyper-plane (bias parameter); and ϕ is the transformation function that converts the input vector ***x*** into some other feature space where the SVM algorithm can provide a linear separation for the training input. By applying the kernel method and duality theorem, the predicted function value for the test input ***x*** can be written as follows:

(3) $$\begin{array}{l} y=\overset{n-1}{\underset{j=1}{\sum }}\alpha_{j}y_{j}\phi {\left(x_{j}\right)}^{\text{T}}\phi \left(x\right)+b=\overset{n-1}{\underset{j=1}{\sum }}\alpha_{j}y_{j}K\left(x_{j},x\right)+b, \end{array}$$

where α_*j*_ is the Lagrange multiplier; and *K*(***x***_*j*_, ***x***) is the kernel function that computes the dot product $\phi {\left(x_{j}\right)}^{\text{T}}\phi \left(x\right)$ and can be generalized to the non-linear case. When the feature dimensionality is high, the data tend to be linearly separable; therefore, we selected the linear kernel other than the commonly used RBF kernel (23). There was only one parameter *C* that controlled the misclassification penalty for the linear SVM model. This parameter was fixed to 1, as previously described (23, 24). When using the linear kernel, the weight vector would be $w=\overset{n-1}{\underset{j=1}{\sum }}\alpha_{j}y_{j}x_{j}$, and the decision function was *y* = *f*(***x***) = ***w***^T^***x*** + *b*, according to Equation (3). According to the SVM optimization theory, the weight vector ***w*** represents the direction along which the feature differed most between two groups. Hence, it was used to produce the discrimination brain map. A positive value in the discrimination map indicated that the patient's FA value was relatively lower than the HC's, whereas a negative value indicated that the HCs had lower FA values. The magnitude of the absolute value of the weight vector ***w*** represented the intensity of between-group differences in FA (25). Given the length of the top ranking features as *k* = *t* × 100, we could obtain the weight vector ***w*** (whose length = *k*) and the bias parameter *b* for each LOOCV fold. Once ***w*** and *b* were calculated, we could predict the function value *y* for test input ***x***. If the function value *y* was >0, then the test subject was assigned to the HC group (label 1); otherwise, the test subject was assigned to the patient group (label 2). The accuracy, sensitivity, and specificity of the classifier model were computed based on these class labels. It should be noted that the mean centering and normalization operations were pre-conducted on the selected features before establishing the SVM model.

As the weight vector ***w*** slightly varied from fold to fold, we calculated the average weight vector to generate the discriminative map. The quantitative analysis of the classifier performance was made by the receiver operating characteristic (ROC), and the permutation test was conducted to evaluate the statistical significance (denoted by *P*-value) of classification accuracy (26, 27). We permuted the class labels of the input subjects randomly and repeated the classification analysis procedure (=10,000 times). We counted the number when the classification accuracy in the permutation test was no less than the real classification accuracy of 83.33%; then, we divided this number by 10,000 to obtain the *P-*value.

In addition, the other DTI-derived parameters (i.e., RD, AD, and MD) were also calculated and analyzed by the above processes. Overall, the machine learning classification performance based on the FA feature was systematically better than those based on the other DTI parameters (Supplementary Table 1 and Supplementary Figures 1–3).

## Results

**Figure 2:**
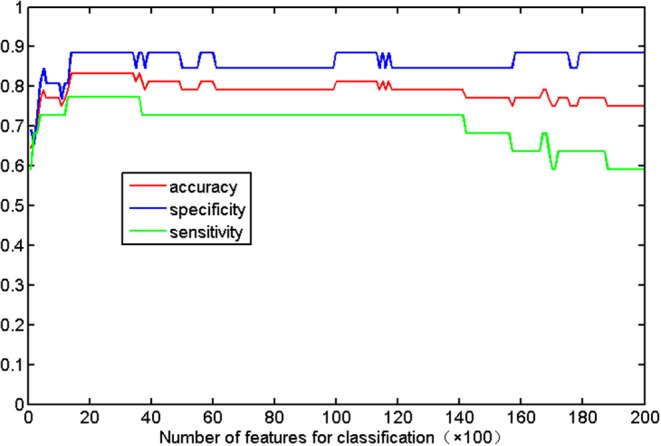
Classification accuracy with respect to the distinct number of FA features. The range of features (from 1,400 to 3,400) can result in the highest accuracy (83.33%), with a sensitivity of 77.27% and specificity of 88.46%.

**Figure 3:**
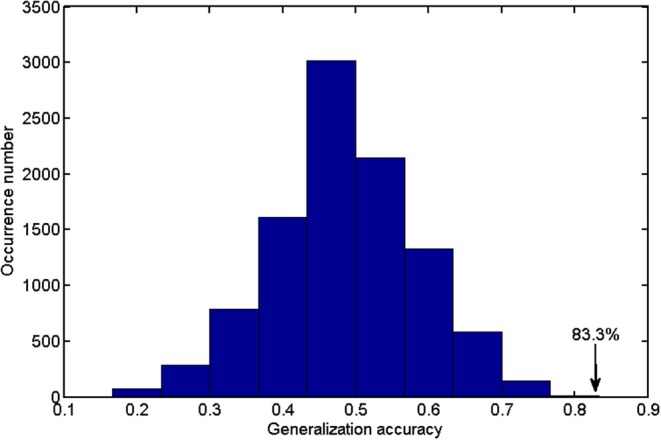
The estimated permutation distribution using the linear SVM classifier (number of repetitions = 10,000), when the 2,400 most representative features were selected. The *x*- and *y*-axes denote the generalization accuracy and occurrence number, respectively. This figure demonstrated that the proposed method was unlikely to exceed the optimal accuracy of 83.33% that was obtained from the real class labels.

Figure 2 shows the detailed results on the accuracy, sensitivity, and specificity based on the distinct number of FA features. When the size of the feature set was small, the input data could not provide enough information to train a reliable model; and when the size was too large, there would be redundant or irrelevant information in the input data, which degraded the machine learning performance. The classification algorithm could attain the optimal performance only when we selected the appropriate dataset. The best classification accuracy was acquired when a specific amount (from 1,400 to 3,400) of features was selected. Thus, we selected the top 2,400 (middle value between 1,400 and 3,400) ranked features as the optimal features. Using these discriminative features, we determined that SVM classifier accuracy can be as high as 83.33% (sensitivity = 77.27% and specificity = 88.46%, *P* = 0.0001). Taking the generalization accuracy as the statistical variable, the estimated permutation distribution is shown in Figure 3, which shows that the probability (when the classification accuracy > 83.33%) is very low (*P* = 0.0001), indicating that our results are highly reliable.

**Figure 4:**
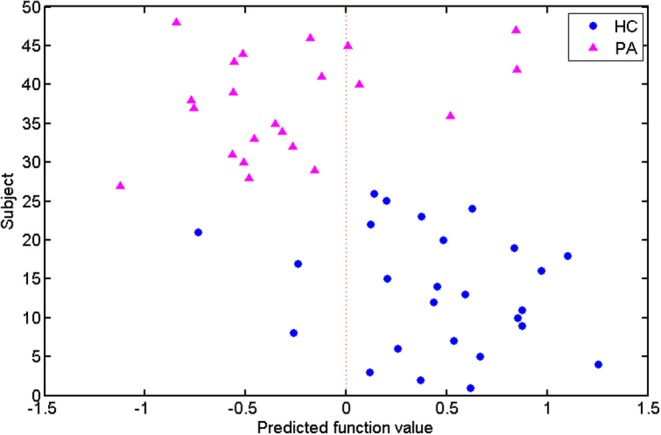
Predicted function values of the test subjects. The healthy controls and patients are indicated by circle and triangle points, respectively. The points on the two sides of the dotted line are labeled as different classes.

In addition, the predicted function value of each test subject was acquired using the SVM classifier (Figure 4). The circle and triangle points represented the HCs and patients, respectively. The circles at the right were the correctly labeled HC subjects, whereas the triangles at the left were correctly labeled patients. According to the distribution of these points, we found that most of the subjects (40/48) were assigned to the correct labels by the classifier.

**Figure 5:**
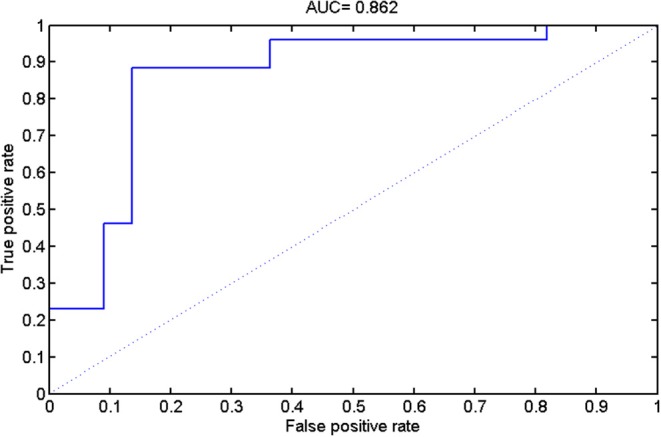
The results of receiver operating characteristic (ROC) curve analysis.

**Figure 6:**
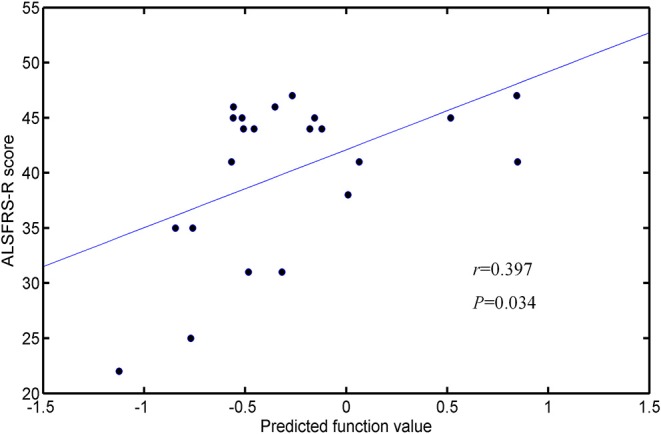
Correlation between the predicted function values and ALSFRS-R scores.

By taking each subject's predicted function value as an indicator, we generated a receiver operating characteristic (ROC) curve for the classifier (Figure 5). The area under the ROC curve (AUC) of the developed method was 0.862, illustrating the relatively strong power for classification. The Spearman correlation coefficient was calculated to assess the correlation between the predicted function value and the ALSFRS-R score (Figure 6). We observed a positive correlation between these parameters (*r* = 0.0.397, *P* = 0.034).

**Figure 7:**
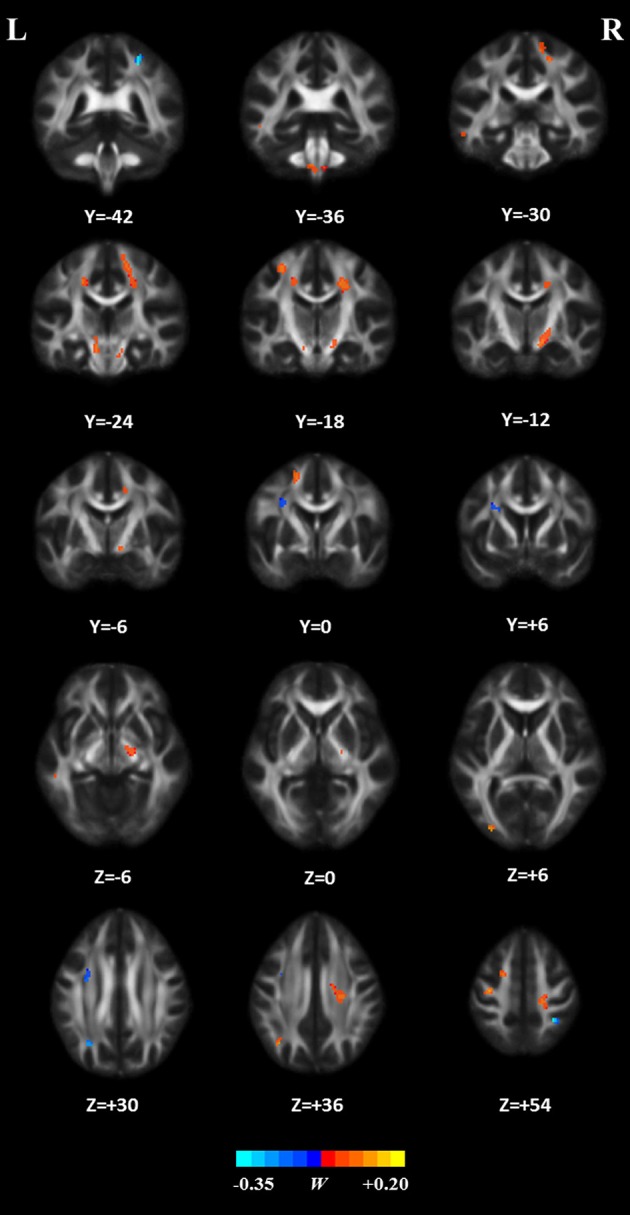
White matter regions whose FA features mostly contribute to classification. These regions were identified by using a threshold of ≥10% of the maximum weight vector score and their cluster size were larger than 20 voxels.

The weight vector ***w*** was used to indicate a subset of features that were most discriminative between the two groups. Thus, the WM regions whose FA value contributed mostly to the discrimination were identified, using a threshold of ≥10% of the maximum weight vector score. The details on these regions are presented in Table 2 and we sorted them according to the contribution for between-group discrimination (i.e., the weight vector ***w***). The relevant distribution of attribute weights generated by SVM analysis is presented in Figure 7. The WM regions with relatively increased FA in the HC group were located in several areas, such as bilateral corona radiate and precentral gyrus, right postcentral gyrus, right posterior limb of internal capsule, left superior frontal gyrus, left angular gyrus, left middle temporal gyrus, left middle occipital gyrus, bilateral midbrain, and bilateral pons and medulla, whereas the WM regions with relatively increased FA in the patient group were located in the left frontal lobe, left inferior parietal lobule, and right superior parietal lobule.

**Table 2:** White matter regions in which the FA feature largely contributed to the classification.

**Cluster size (number of voxels)**	**White matter region**	**MNI coordinates**			**Peak W*i***
		**x**	**y**	**z**	
**HC group > ALS group**					
31	Left middle occipital gyrus	−24	−84	10	0.158
23	Left angular gyrus	−34	−62	38	0.146
39	Left precentral gyrus	−30	−14	56	0.132
297	Right corona radiate/precentral and postcentral gyrus	22	−20	40	0.120
27	Left superior frontal gyrus	−18	0	54	0.106
125	Right midbrain and posterior limb of internal capsule	12	−12	−12	0.105
48	Left midbrain	−8	−26	−18	0.099
58	Left corona radiate	−22	−22	42	0.095
46	Bilateral pons and medulla	−6	−34	−40	0.089
20	Left middle temporal gyrus	−54	−34	−12	0.018
**HC group < ALS group**					
27	Right superior parietal lobule	26	−42	52	−0.335
21	Left inferior parietal lobule	−28	−64	28	−0.241
51	Left frontal lobe	−30	−2	32	−0.137

*The above brain regions were identified by using a threshold of ≥10% of the maximum weight vector score. The first column only lists clusters larger than 20 voxels. Wi (reported in the last column) is the weight of each cluster centroid, i.e., the value that indicates the relative contribution of the FA feature to the SVM-based classification*.

## Discussion

In the present study, we combined DTI with SVM to classify ALS patients and HCs. The high classification accuracy of 83.33% can be obtained in the optimized SVM model. The permutation statistics further validated the reliability of our SVM classifier. The FA values from both motor and extra-motor areas contributed to the classification, which may indicate that ALS is a multi-system neurodegenerative disease. Further ROC analysis also indicated the high potential of FA measurement in the accurate discrimination of ALS. Moreover, the predicted function value of classifier was correlated with ALS disease severity. These results suggested the promising perspective related to the application of SVM approach to ALS identification, based on the DTI measurement in WM.

The impairments of WM integrity (as reflected by reduced FA) have been well-documented in ALS, which may be due to the destruction of the axons and myelin (6, 28, 29). An early neuropathological feature of ALS is defective axonal transport, which may contribute to distal axon energy deficiency and dying-back axonopathy (30, 31). Oligodendrocytes myelinate the central nervous system (CNS) axons and support the function and survival of axons (32, 33). The pathological abnormalities in oligodendrocytes (e.g., oligodendrocyte death and impaired maturation of new oligodendrocytes) have been reported in ALS (34, 35), which could contribute to axonal demyelination (36).

Consistent with previous studies (37, 38), we found that the WM regions with decreased FA in ALS involved the bilateral precentral gyrus and the CST pathway, such as bilateral corona radiate, right posterior of internal capsule, bilateral midbrain, and bilateral pons, and medulla. The precentral gyrus is part of the primary motor cortex (PMC). The degenerative alterations of the PMC such as significantly decreased Betz cells and cortical thinning (39, 40) have been reported in ALS. The CST, which connects the neurons in the motor cortex and spinal cord, is the fibers that are associated with cortical control of spinal cord activity (41). Degeneration of the CST is also a hallmark of ALS (42). In sum, damage to these motor-related regions could lead to motor neuron dysfunction and is responsible for the relevant symptoms (e.g., muscle weakness and loss of voluntary control) observed in ALS patients (43).

The regions with decreased FA also included several extra-motor areas such as the right postcentral gyrus, left superior longitudinal fasciculus (SLF) that involves the left superior frontal gyrus, left angular gyrus, and left middle temporal gyrus, and left inferior longitudinal fasciculus (ILF) that involves the left middle temporal gyrus and left middle occipital gyrus, which agrees with the findings of previous studies (44–46). For example, it has been demonstrated that the significant cortical thinning of the postcentral gyrus, namely, primary somatosensory cortex (47), occurring in ALS is correlated with disease severity (48, 49). In addition, the SLF, which connects the frontal, parietal, and temporal lobes and plays a key role in language function (50, 51), is disrupted in ALS (52, 53). Meanwhile, damage to the left ILF, the fiber that is primary associated with visual processing, language/semantic function, and regulation of emotion (54, 55), has been reported in ALS patients (45, 56). Therefore, damage to these extra-motor regions, as reflected by decreased FA, may be associated with the non-motion dysfunctions that have been reported in ALS, such as sensory deficits, language dysfunction, and behavioral and psychiatric abnormalities (57–59).

In contrast, our results showed several brain areas, including the left frontal lobe, left inferior parietal lobule, and right superior parietal lobule, with relatively increased FA in ALS patients. It is speculated that these changes may be related to the functional compensation or the reorganization of the cerebral structure in ALS (60, 61). In agreement with this speculation, several compensatory phenomena, as reflected by the increased gray matter volume or the overactivation of specific brain regions (62, 63), have been demonstrated in ALS.

The accurate recognition and characterization of ALS remain challenging due to its low incidence [≈5/100,000 (64)] and heterogeneous nature (1, 15). Our results suggest that DTI measurement in the white matter could be utilized as an alternative biomarker of ALS based on machine learning method. Consistently, previous studies have also demonstrated the potential of diffusion measurement in identifying ALS, at the group and individual levels (6, 15, 16, 65, 66). The ROC analysis and permutation statistics further verified the reliability of our classification results. Moreover, our results suggested that FA was the most promising biomarker for ALS identification, relative to other DTI metrics, which keeps in line with previous evidences highlighting FA change is the consistent hallmarker of ALS (6). In addition, the correlation between the SVM predicted function value and the ALSFRS-R score was observed. As the predicted function value was computed by projecting the optimal features onto the weight vector of the hype-plane, a larger absolute value meant that the subject was situated farther away from the hyper-plane and more significantly contributed to the classification. Thus, we could deduce that when the ALSFRS-R score was higher (i.e., higher disease severity), the subject is less likely to be misclassified.

From a data-driven methodological perspective, this study employed SVM method to establish the predicting model of ALS, given that SVM can accommodate all of voxel-wise DTI measures simultaneously and can model their interactions in high dimensional feature space to optimize between-group classification. As a supervised learning model, the advantage of SVM relies on its regularization parameter that is helpful in preventing model overftting and SVM also have good performance and generalization capability when processing small-sample data (67, 68), such that SVM formalism was preferred for this exploratory work with the limited sample size. Different from our study, recent machine learning researches using DTI metrics of the pre-defined region of interest (e.g., CST) have applied Random Forests approach to build discrimination model between ALS and healthy control (10–12). Random Forests holds the advantages over other methods in the aspects of ability to handle highly non-linear biological data, robustness to noise, and tuning simplicity (12, 69). Taken together, it is noted that SVM and other methods (such as Random Forests) can be used in the implementation of prediction modeling of ALS and show the distinct methodological advantages, thereby, a systemic comparison of the diagnostic performances of various machine learning methods is recommended in the future.

This study has a number of limitations. First, unlike other previous studies on ALS, we did not consider disease heterogeneity (70, 71) and did not perform any investigation to examine the potential of DTI measurements in distinguishing ALS subtypes. Second, despite the prevalence of the SVM algorithm for medical data analysis, other machine learning algorithms could also be explored to promote the performance of the classification model and seek more reliable biomarkers for ALS patients. Third, other neuroimaging features (e.g., functional connectivity) can also contribute to ALS identification (17), so the combination of DTI and other modalities of MRI data should be considered in future classification studies. Fourth, further investigations using a larger cohort are recommended to further validate the findings of our study.

Our results suggest the feasibility of ALS diagnosis based on SVM analysis and diffusion measurements of WM. The WM regions whose FA values mostly contributed to SVM classification involved motor as well as extra-motor areas, thereby supporting the notion that ALS is a disease involving multi-system neurodegeneration. In addition to the existing studies, our findings further provided the confirmatory evidences that the application of machine learning method such as SVM with neuroimaging data holds the promising perspective for the prediction of ALS. However, in consideration of the small sample size and other limitations, this study represented an exploratory work in nature. The further investigation using a larger cohort is warranted to validate the generality of our results and the future studies are recommended to verify the added diagnostic value of the inclusion of other modality neuroimaging data.

## Data Availability Statement

The original contributions presented in the study are included in the article/Supplementary Files, further inquiries can be directed to the corresponding author.

## Ethics Statement

The studies involving human participants were reviewed and approved by Approval for this evaluation was obtained from the Research Ethics Committee of Fujian Medical University Union Hospital, China. All of the subjects provided their written informed consent. The patients/participants provided their written informed consent to participate in this study.

## Author Contributions

H-JC, Q-FC, and X-HZ conceived and designed the study, acquired and analyzed the data, and wrote the manuscript. H-JC, Q-FC, and N-XH contributed to data analysis. All authors have read and approved the manuscript.

## Conflict of Interest

The authors declare that the research was conducted in the absence of any commercial or financial relationships that could be construed as a potential conflict of interest.

## Abbreviations

ALS: amyotrophic lateral sclerosis
ALSFRS-R: revised ALS Functional Rating Scale
DTI: diffusion tensor imaging
FA: fractional anisotropy
AD: axial diffusivity
RD: radial diffusivity
MD: mean diffusivity
SVM: support vector machine
ROC: receiver operating characteristic
LOOCV: leave-one-out cross-validation
CST: corticospinal tract
SLF: superior longitudinal fasciculus
ILF: inferior longitudinal fasciculus.
