# Supplementary figures and images for: Identification of Amyotrophic Lateral Sclerosis Based on Diffusion Tensor Imaging and Support Vector Machine
Source: Front Neurol. 2020 Apr 28;11:275. doi: 10.3389/fneur.2020.00275 (PMC7198809; doi:10.3389/fneur.2020.00275)

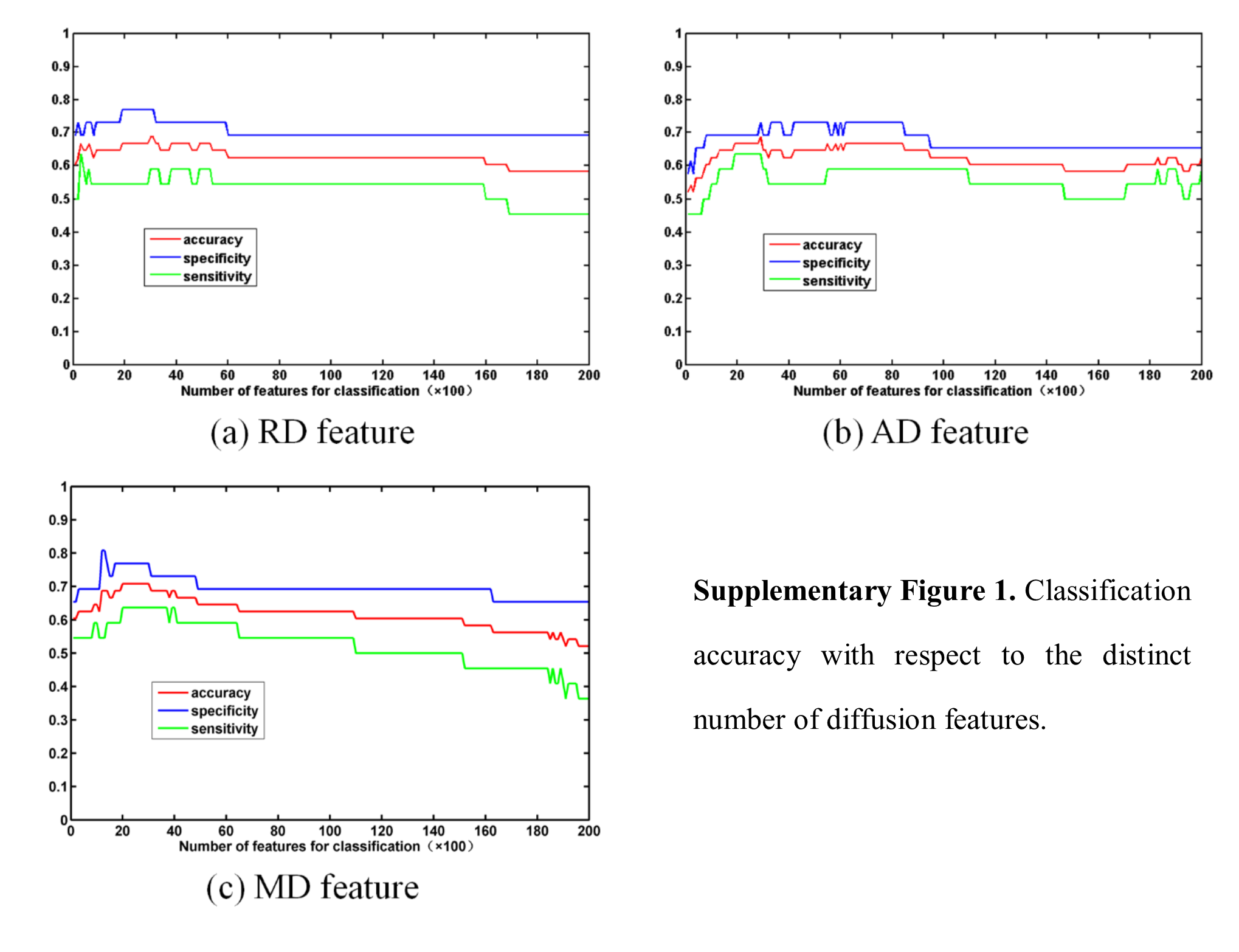

Supplement: Supplementary file 1 [file Image_1.tif]

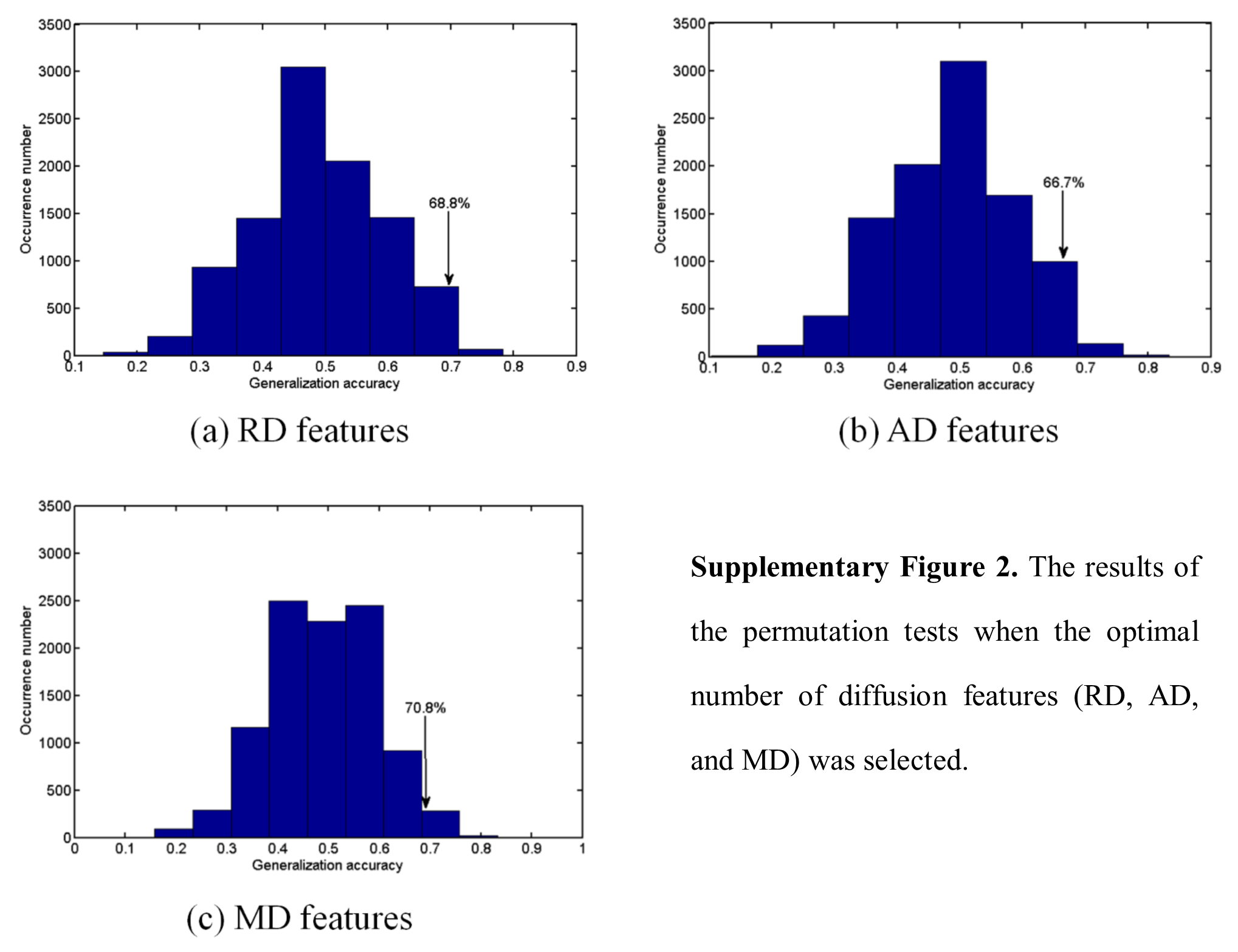

Supplement: Supplementary file 2 [file Image_2.tif]

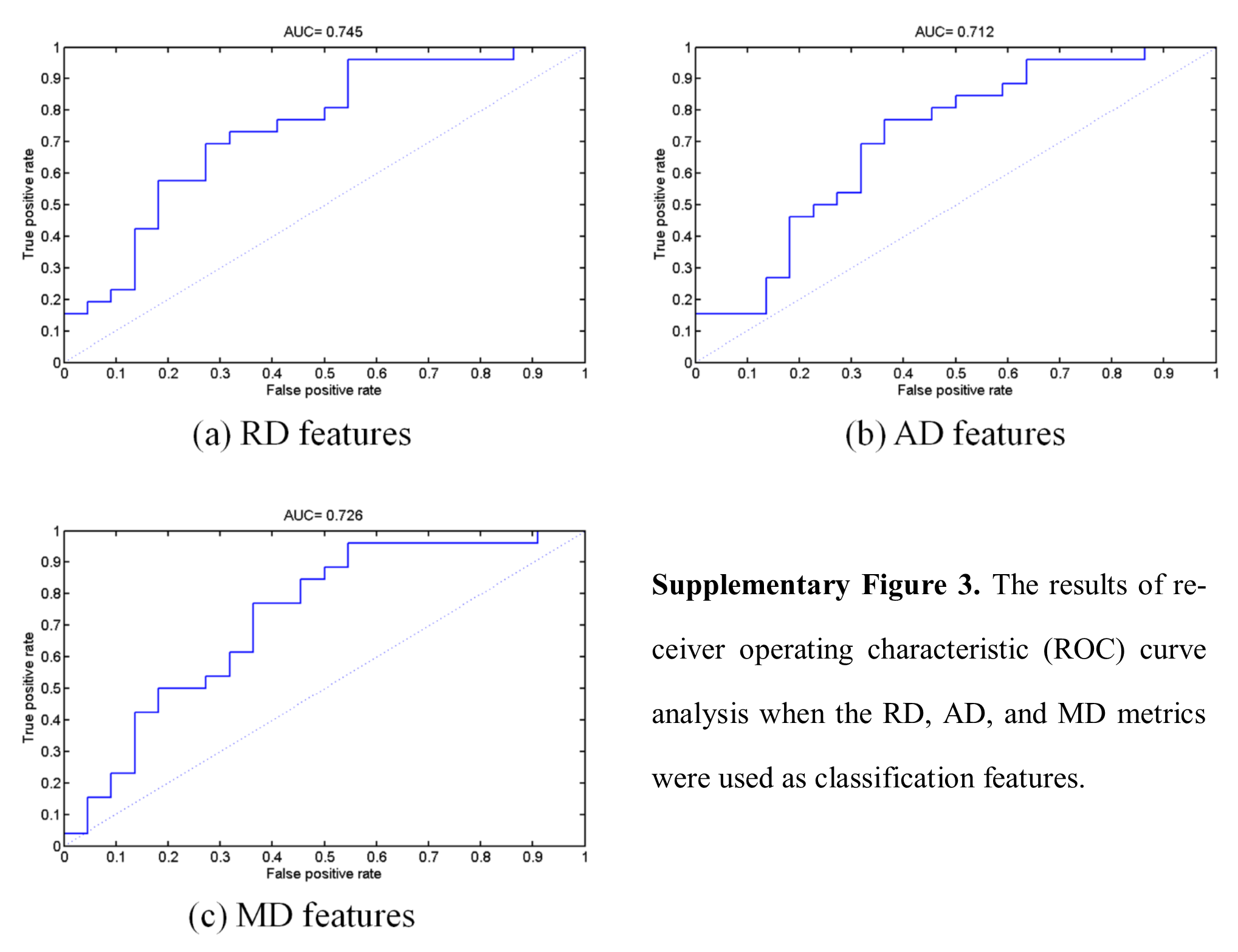

Supplement: Supplementary file 3 [file Image_3.tif]
