# Supplementary material for: Identification of Amyotrophic Lateral Sclerosis Based on Diffusion Tensor Imaging and Support Vector Machine
Source: Front Neurol. 2020 Apr 28;11:275. doi: 10.3389/fneur.2020.00275 (PMC7198809; doi:10.3389/fneur.2020.00275)
Supplement: Supplementary file 4 [file Table_1.pdf]

Supplementary Table 1. The classification results based on other diffusion features

	RD	AD	MD
Range of feature number (when the highest classification accuracy was achieved)	3,000~3,100	1,900~2,800	2,000~3,000
Optimal feature number	3,000	2,400	2,500
Accuracy	68.75%	66.67%	70.83%
Specificity	76.92%	69.23%	76.92%
Sensitivity	59.09%	63.64%	63.64%
AUC	0.745	0.712	0.726
P value in permutation test	0.0152	0.0474	0.0163

RD, radial diffusivity; AD, axial diffusivity; MD, mean diffusivity
